# *Hisonotus acuen*, a new and phenotypically variable cascudinho (Siluriformes, Loricariidae, Hypoptopomatinae) from the upper rio Xingu basin, Brazil

**DOI:** 10.3897/zookeys.442.7870

**Published:** 2014-09-25

**Authors:** Gabriel S. C. Silva, Fábio F. Roxo, Claudio Oliveira

**Affiliations:** 1 Universidade Estadual Paulista, Departamento de Morfologia, Laboratório de Biologia e Genética de Peixes, Botucatu, SP, Brazil

**Keywords:** Biodiversity, Cascudinhos, Fresh-water, Neotropical fish, Taxonomy

## Abstract

A new species of *Hisonotus* is described from the headwaters of the rio Xingu. The new species is distinguished from its congeners by having a functional V-shaped spinelet, odontodes not forming longitudinal aligned rows on the head and trunk, lower counts of the lateral and median series of abdominal figs, presence of a single rostral fig at the tip of the snout, absence of the unpaired figlets at typical adipose fin position, yellowish-tipped teeth, absence of conspicuous dark saddles and stripe on the body and higher number of teeth on the premaxillary and dentary. The new species, *Hisonotus acuen*, is restricted to headwaters of the rio Xingu basin, and is the first species of the genus *Hisonotus* described from the rio Xingu basin. *Hisonotus acuen* is highly variable in aspects of external body proportions, including body depth, snout length, and abdomen length. This variation is partly distributed within and among populations, and is not strongly correlated with body size. PCA of 83 adult specimens from six allopatric populations indicates the presence of continuous variation. Therefore, the available morphological data suggest that the individuals inhabiting the six localities of rio Xingu represent different populations of a single species. Low intraspecific variation in mitochondrial Cytochrome oxidase subunit I (COI) provides corroborative evidence.

## Introduction

The subfamily Hypoptopomatinae is a monophyletic group of Loricariids (Schaefer, 2003) composed of 19 genera and 139 species (Eschmeyer and Fong 2014). Within this subfamily, *Hisonotus* Eigenmann & Eigenmann, 1889 comprises 33 valid species (Eschmeyer 2014). The genus *Hisonotus* was resurrected from the synonymy of *Otocinclus* by Schaefer (1998) based on the reduced or absent snout figs anterior to the nostril, rostrum with enlarged odontodes, and thickened figs forming the lateral rostral margin. However, the phylogenetic relationships in this genus are not well resolved (Britski and Garavello 2007) and, according to molecular (Chiachio et al. 2008; Cramer et al. 2011) and morphological (Martins et al. 2014) data, *Hisonotus* is a polyphyletic genus.

Although there is no definition of *Hisonotus* that supports its monophyly, many authors have considered this genus as valid. In the past decade, 18 species of *Hisonotus* have been described (Britski and Garavello 2007; Carvalho et al. 2008; Carvalho and Reis 2009; Carvalho and Reis 2011; Martins and Langeani 2012; Carvalho and Datovo 2012; Roxo et al. 2013; Roxo et al. 2014). Recently, during a collecting trip in tributaries of the rio Xingu basin, we found fish specimens that have the generally accepted characteristics of *Hisonotus* listed above but do not match any known species. Herein we describe the rio Xingu specimens as a new species.

## Material and methods

All measurements and counts were taken on the left side of specimens. Measurements were taken point to point to the nearest 0.1 mm with a digital caliper. Body fig and osteology nomenclature followed Schaefer (1997) and measurements followed Carvalho and Reis (2009), as shown in Table 1. Abbreviations used in the text followed Carvalho and Reis (2009). Morphometrics are given as percentages of standard length (SL), except for subunits of the head region, which are expressed as percentages of head length (HL). Specimens were cleared and stained (c&s) according to the method of Taylor and Van Dyke (1985). Vertebral counts also include the five vertebrae that comprise the Weberian apparatus. Dorsal-fin ray counts include the spinelet as the first unbranched ray. All examined specimens were collected according to the Brazilian laws, and are deposited under permanent scientific collection licenses. After collection, specimens were euthanized using 1% benzocaine in water, fixed in 10% formaldehyde for morphological studies and preserved in 70% alcohol. For molecular studies specimens were fixed directly in 95% alcohol. Sequencing and molecular analysis followed Roxo et al. (2012). Institutional acronyms follow Fricke and Eschmeyer (2014). All samples are deposited at the LBP, Laboratório de Biologia e Genética de Peixes, Universidade Estadual Paulista, Botucatu; MZUSP, Museu de Zoologia, Universidade de São Paulo, São Paulo; NUP, Coleção Ictiológica do Nupelia, Universidade Estadual de Maringá, Maringá. Zoological nomenclature follows the International Code of Zoological Nomenclature (4th Ed.). The GenBank accession numbers for Cytochrome oxidase subunit I (COI) sequences are: *Hisonotus acuen* – KM365043, KM365044, KM365045, KM365046, KM365047, KM365048, KM365049, KM365050, KM104473; *Hisonotus chromodontus* – KM104474, KM104475, JN998567, JN998566, JN998567, JN998565, JN998564, KM365054; *Hisonotus insperatus* – KM104485, GU701888, GU701749, GU701748, GU701747, GU701746, KM365055, KM365056, KM365057, KM365058, KM365059, KM365060, KM365061; *Hisonotus notatus* – JN998579, JN998581, JN998580; *Hisonotus oliveirai* – KM104486, KM365062, KM365063; *Hisonotus paresi* – KM104490, KM365042; *Hisonotus piracanjuba* – KM104487, KM104488, KM365051, KM365052, KM365053.

**Table 1.** Morphometrics and meristic data for all analyzed specimens of *Hisonotus acuen* and for specimens by sub basins. SD = Standard deviation.

	*Hisonotus acuen*, n = 83					rio Toguro, n = 7				rio Culuene, n = 29				rio Suiá Missu, n = 15			
	Holotype	Low	High	Mean	SD	Low	High	Mean	SD	Low	High	Mean	SD	Low	High	Mean	SD
SL	25.9	18.2	29.0	23.3	2.6	20.5	25.9	22.9	2.23	21.7	29.0	25.6	1.7	20.5	27.1	23.3	2.0
**Percents of SL**																	
Head length	39.4	35.1	44.1	39.4	1.8	38.4	44.1	40.7	1.7	8.81	10.8	9.7	0.4	8.3	10.5	9.3	0.6
Predorsal length	50.1	41.9	54.3	50.0	1.9	48.6	52.8	51.1	1.5	41.9	54.3	48.8	2.0	49.1	52.7	51.1	1.1
Dorsal-fin spine length	20.6	19.4	25.8	21.9	1.4	19.5	22.8	20.7	1.06	4.9	6.3	5.6	0.3	4.0	6.2	5.0	0.6
Anal-fin unbranched ray length	15.3	13.7	19.9	16.9	1.3	14.9	16.6	15.7	0.69	3.8	5.0	4.4	0.2	2.8	4.2	3.6	0.3
Pectoral-fin spine length	24.0	15.9	28.8	25.0	1.9	21.6	26.6	23.9	1.68	22.7	28.8	25.6	1.4	4.9	6.6	5.8	0.5
Pelvic-fin unbranched ray length	16.1	13.1	25.0	16.9	2.1	13.9	17.6	15.8	1.17	15.2	23.2	17.6	2.1	2.8	4.1	3.6	0.3
Cleithral width	22.1	13.2	27.5	23.9	1.9	22.1	24.6	23.6	1.05	5.2	7.0	6.3	0.5	4.6	6.2	5.4	0.5
Thoracic length	12.8	10.5	23.1	13.7	2.0	11.2	16.5	13.4	2.07	2.8	4.5	3.7	0.4	2.2	5.8	3.2	0.9
Abdominal length	22.0	10.2	24.4	20.8	2.5	18.3	22.0	20.6	1.16	3.6	6.6	5.6	0.6	2.1	6.0	4.5	1.1
Caudal-peduncle length	27.7	25.5	33.0	28.6	1.5	26.1	30.0	27.7	1.18	6.3	8.6	7.4	0.6	5.2	7.8	6.5	0.7
Caudal-peduncle depth	9.5	8.6	11.1	9.6	0.4	8.7	10.3	9.5	0.54	8.9	11.1	9.9	0.4	9.0	10.3	9.5	0.4
**Percents of HL**																	
Snout length	56.0	34.2	57.2	53.5	2.6	41.2	56.0	51.8	4.95	51.5	56.7	53.6	1.52	34.2	56.4	53.0	5.4
Orbital diameter	13.4	11.2	16.2	13.1	0.9	11.6	13.8	12.7	0.83	11.7	15.0	13.4	0.8	1.0	1.3	1,1	0.1
Interorbital width	36.2	15.6	41.8	36.3	3.4	33.1	53.0	37.1	1.05	3.1	4.1	3.7	0.2	2.7	3.9	3.3	0.3
Head depth	51.7	35.1	53.1	44.2	4.6	40.0	56.5	45.5	6.1	3.9	5.3	4.6	0.3	3.2	4.8	4.0	0.5
Suborbital depth	19.2	13.3	22.4	17.4	2.2	12.6	19.2	16.4	2.3	16.0	21.0	19.0	1.3	1.1	1.8	1.5	0.2
Mandibular ramus	11.5	6.9	12.9	10.2	1.4	9.7	17.9	12.5	3.6	8.3	12.9	10.9	1.3	8.5	9.6	9.1	0.4
**Meristics**																	
Left premaxillary teeth	23	14	27	14	4.1	14	20	-	3.87	22	24	23.3	0.8	14	21	17.2	2.5
Left dentary teeth	18	12	23	21	3.5	13	18	-	2.38	12	23	21	3.5	13	17	15.0	1.5
Left lateral scutes	23	22	24	23	0.7	23	24	23	0.82	20	23	21.3	1.0	24	24	24	-

	córrego Xavante, n = 13				rio Coronel Vanick, n = 4				rio Feio, n = 15			
	Low	High	Mean	SD	Low	High	Mean	SD	Low	High	Mean	SD
SL	19.4	25.6	22.1	2.0	19.5	24.8	21.5	2.4	18.2	24.6	20.8	1.9
**Percents of SL**												
Head length	37.2	41.7	39.1	1.2	38.2	41.2	39.9	1.3	39.2	42.7	41.2	1.1
Predorsal length	47.5	51.5	49.2	1.1	49.1	52.9	51.1	1.6	49.0	52.9	51.3	1.3
Dorsal-fin spine length	20.2	24.7	22.2	1.1	19.4	21.6	20.5	0.7	20.0	24.4	22.3	1.1
Anal-fin unbranched ray length	16.4	19.9	17.6	0.8	13.9	16.7	15.7	1.2	15.5	19.7	17.7	1.0
Pectoral-fin spine length	15.9	27.7	24.5	3.6	23.2	25.6	24.8	1.0	23.3	26.8	24.9	1.0
Pelvic-fin unbranched ray length	15.5	20.7	17.8	1.7	15.6	16.3	15.9	0.3	13.1	25.0	16.8	2.8
Cleithral width	23.9	27.5	25.5	0.8	13.2	25.4	22.0	5.8	20.8	22.9	21.8	0.6
Thoracic length	10.5	16.6	12.8	1.5	11.6	13.5	12.7	0.8	11.2	19.0	12.9	2.0
Abdominal length	12.1	22.5	20.3	2.5	20.3	22.1	21.2	0.7	18.4	23.9	20.3	1.3
Caudal-peduncle length	27.1	32.1	29.2	1.3	26.2	27.6	26.7	0.6	26.8	30.9	28.8	1.4
Caudal-peduncle depth	9.1	10.3	9.7	0.3	9.1	9.7	9.4	0.2	8.6	9.9	9.2	0.3
**Percents of HL**												
Snout length	50.3	54.6	52.9	1.1	51.3	53.7	52.7	1.0	52.7	57.2	54.9	1.24
Orbital diameter	12.2	16.2	13.7	1.1	12.5	14.2	13.4	0.7	11.2	14.6	13.1	0.9
Interorbital width	35.8	41.8	38.5	1.6	33.8	36.8	35.1	1.2	15.6	37.6	32.9	5.1
Head depth	40.6	51.4	46.5	3.0	40.2	43.8	41.6	1.5	35.1	39.9	37.5	1.5
Suborbital depth	14.7	22.4	19.1	2.0	14.4	18.4	16.1	1.9	13.3	17.5	14.9	1.2
Mandibular ramus	7.3	10.1	8.8	1.0	7.2	8.9	8.1	0.4	6.9	11.5	9.3	1.2
**Meristics**												
Left premaxillary teeth	20	27	-	2.94	17	25	-	4.0	14	15	14	0.5
Left dentary teeth	16	22	-	2.75	14	17	-	1.5	12	13	12	0.5
Left lateral scutes	23	24	23	0.5	23	24	23	0.5	22	24	22	1.5

### Principal component analysis (PCA)

Principal component analysis (PCA) was used to check overall variation among samples, including differences in morphometrics among species. PCA is a statistical procedure that uses orthogonal transformation to convert a set of observations of possibly correlated variables into a set of values of linearly uncorrelated variables called principal components (Jolliffe 2002). The analyses were made using all measurements listed above. Juvenile specimens below 18.0 mm SL were excluded from the analyses. PCA on covariances of base 10 logarithmically transformed measurements to reduce the influence of size were obtained using Past version 1.28 (Hammer et al. 2004). The PCA Loadings are presented in Table 2.

**Table 2.** Variable loadings in the first and second axes of size-free Principal Component Analysis (Axis 1 and Axis 2) of combined samples of *Hisonotus acuen*.

	Axis 1	Axis 2
Predorsal length	0.1871	-0.0346
Preanal length	0.2139	-0.0923
Head length	0.1631	-0.0090
Cleithral width	0.2370	0.1401
Dorsal-fin spine length	0.2015	0.0902
Base of dorsal-fin length	0.2838	-0.2170
Thorax length	0.2649	-0.1924
Pectoral-fin spine length	0.2234	0.0939
Abdomen length	0.2688	-0.5397
Pelvic-fin spine length	0.2155	0.4822
Anal-fin spine length	0.1747	0.2453
Lower cd spine	0.2088	0.0789
Caudal peduncle depth	0.1887	0.0911
Caudal peduncle length	0.2168	0.3770
Anal width	0.2543	-0.1638
Body depth	0.2608	-0.2413
Head depth	0.2506	0.0613
Snout length	0.1376	-0.1066
Interorbital width	0.2147	0.1441
Orbital diameter	0.1189	0.0778
Suborbital depth	0.2368	0.0426

## Results

### Hisonotus acuen sp. n.

Taxon classificationAnimaliaSiluriformesLoricariidae

http://zoobank.org/12454B4D-E9CA-4A89-A307-B77A703308C9

**Figure 1.**
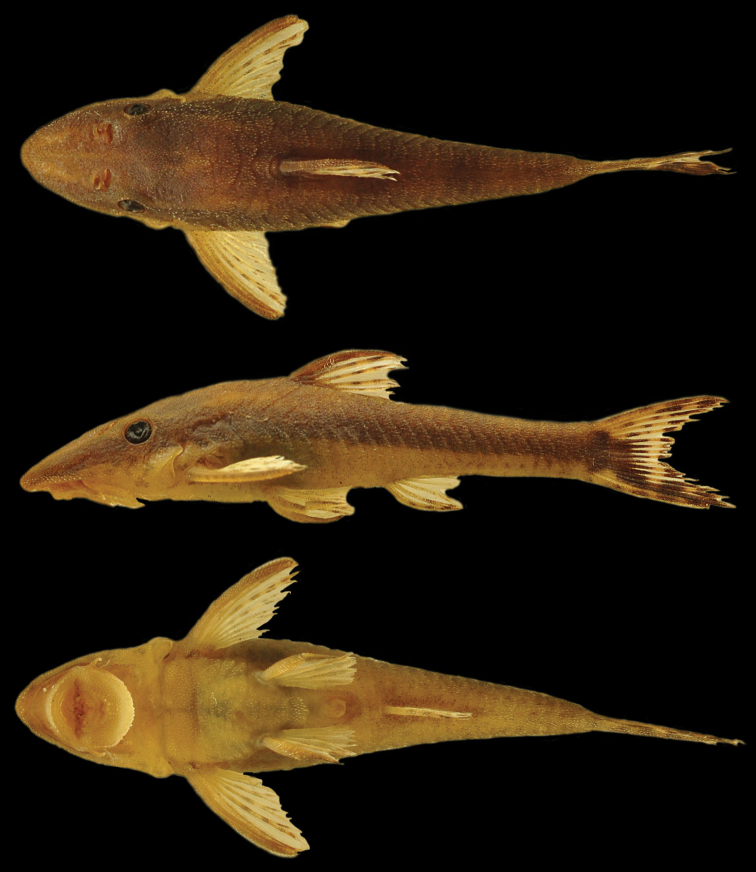
*Hisonotus acuen*, MZUSP 115350, female, 25.9 mm SL, holotype, from Mato Grosso State, municipality of Querência, affluent of rio Toguro, rio Xingu basin, 13°00'26"S, 52°11'27"W.

Figs 1 , 5 , 6 ; Table 1

#### Holotype.

MZUSP 115350, female, 25.9 mm SL, Brazil, Mato Grosso State, municipality of Querência, affluent of rio Toguro, rio Xingu basin, 13°00'26"S, 52°11'27"W, 01 Aug 2012, coll. C. Oliveira, M. Taylor, G.J.C. Silva & J.M. Henriques.

#### Paratypes.

All from Brazil, Mato Grosso State, rio Xingu basin. LBP 15755, 16, 19.5-26.0 mm SL, municipality of Ribeirão Cascalheira, affluent of rio Suiá-Missu, 12°55'36"S, 51°53'27"W, 30 July 2012, coll. Oliveira C, Taylor M, Silva GJC, Henriques JM. LBP 16274, 27, 20.2–29.1 mm SL, 2 c&s 23.6−24.2 mm SL, municipality of Gaúcha do Norte, affluent of rio Culuene, 13°27'26"S, 53°09'36"W, 03 Aug 2012, coll. Oliveira C, Taylor M, Silva GJC, Henriques JM. LBP 16275, 29, 16.7-25.2 mm SL, 2 c&s 19.3−20.8 mm SL, municipality of Querência, affluent of rio Feio, 12°33'20"S, 52°16'16"W, 31 Sep 2012, coll. Oliveira C, Taylor M, Silva GJC, Henriques JM. LBP 16276, 9, 20.7-27.9 mm SL, 2 c&s 21.2−21.4 mm SL, municipality of Ribeirão Cascalheira, affluent of rio Suiá-Missu, 12°53'04"S, 52°02'00"W, 30 Sep 2012, coll. Oliveira C, Taylor M, Silva GJC, Henriques JM. LBP 16277, 10, 18.9–23.3 mm SL, municipality of Querência, affluent of rio Feio, 12°31'55"S, 52°20'29"W, 31 Sep 2012, coll. Oliveira C, Taylor M, Silva GJC, Henriques JM. LBP 16278, 12, 18.8–25.1 mm SL, 2 c&s 26.8−27.1 mm SL, municipality of Primavera do Leste, córrego Xavante, 14°38'24"S, 53°55'38"W, 05 Aug 2012, coll. Oliveira C, Taylor M, Silva GJC, Henriques JM. LBP 16279, 10, 20.8-26.7 mm SL, municipality of Gaúcha do Norte, affluent of rio Culuene, 13°26'32"S, 53°08'45"W, 03 Aug 2012, coll. Oliveira C, Taylor M, Silva GJC, Henriques JM. LBP 16280, 11, 17.4–24.9 mm SL, municipality of Canarana, affluent of rio Culuene, 13°25'30"S, 52°16'47"W, 01 Aug 2012, coll. Oliveira C, Taylor M, Silva GJC, Henriques JM. LBP 16281, 4, 17.5–24.6 mm SL, same collection information as holotype. LBP 16282, 5, 17.5-23.9 mm SL, municipality of Canarana, rio Coronel Vanick, 13°31'34"S, 52°43'52"W, 02 Aug 2012, coll. Oliveira C, Taylor M, Silva GJC, Henriques JM. LBP 16283, 2, 21.3-24.2 mm SL, municipality of Canarana, affluent of rio Toguro, 13°16'52"S, 52°14'42"W, 01 Aug 2012, coll. Oliveira C, Taylor M, Silva GJC, Henriques JM. LBP 16284, 3, 20.2–24.3 mm SL, collected with holotype. LBP 18845, 1, 23.7 mm SL, municipality of Gaúcha do Norte, affluent of rio Culuene, 13°30'57"S, 53°06'39"W, 03 Aug 2012, coll. Oliveira C, Taylor M, Silva GJC, Henriques JM. NUP 16444, 5, 22.2–27.1 mm SL, municipality of Gaúcha do Norte, affluent of rio Culuene, 13°27'26"S, 53°09'36"W, 03 Aug 2012, coll. Oliveira C, Taylor M, Silva GJC, Henriques JM.

#### Diagnosis.

**Figure 2.**
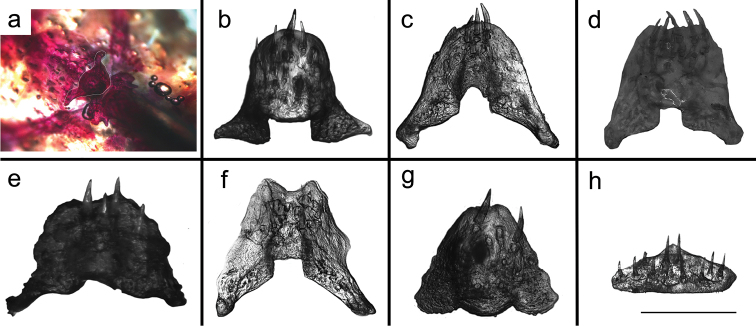
Spinelet variation among *Hisonotus* species. Figure a-f showing the extension of the bone forming a lock mechanism. In figure **g–h**, the bones lack the lock mechanism: **a** *Hisonotus acuen*, LBP 16276, 21.4 mm SL **b** *Hisonotus acuen*, LBP 16274, 21.1 mm SL **c** *Hisonotus oliveirai* LBP 13332, 23.7 mm SL **d** *Hisonotus chromodontus*, LBP 7964, 28.3 mm SL **e** *Hisonotus piracanjuba*, LBP 17256, 27.1 mm SL **f** *Hisonotus paresis*, NUP 10928, 23.6 mm SL **g** Hisonotus cf. notatus, LBP 3472, 25.8 mm SL **h** *Hisonotus depressicauda*, LBP 17474, 28.1 mm SL. Scale bar = 0.5 mm.

**Figure 3.**
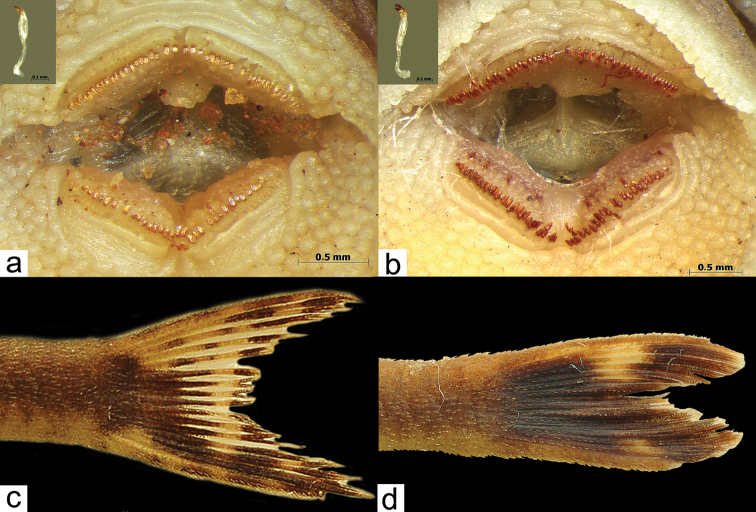
**a** *Hisonotus acuen*, holotype, MZUSP 115350, 25.9 mm SL, showing the yellowish-tipped teeth **b** *Hisonotus chromodontus*, NUP 10924, 29.7 mm SL, showing the reddish-brown teeth **c** *Hisonotus acuen*, holotype, MZUSP 115350, 25.9 mm SL, showing the caudal-fin color pattern mostly hyaline, except for dark blotch on origin of rays, and dark brown chromatophores largely concentrated on rays near lower caudal spine **d** *Hisonotus chromodontus*, LBP7964, 27.3 mm SL, showing caudal-fin dark brown with chromatophores largely concentrated on rays and membranes, and with two hyaline spots on middle of the fin.

**Figure 4.**
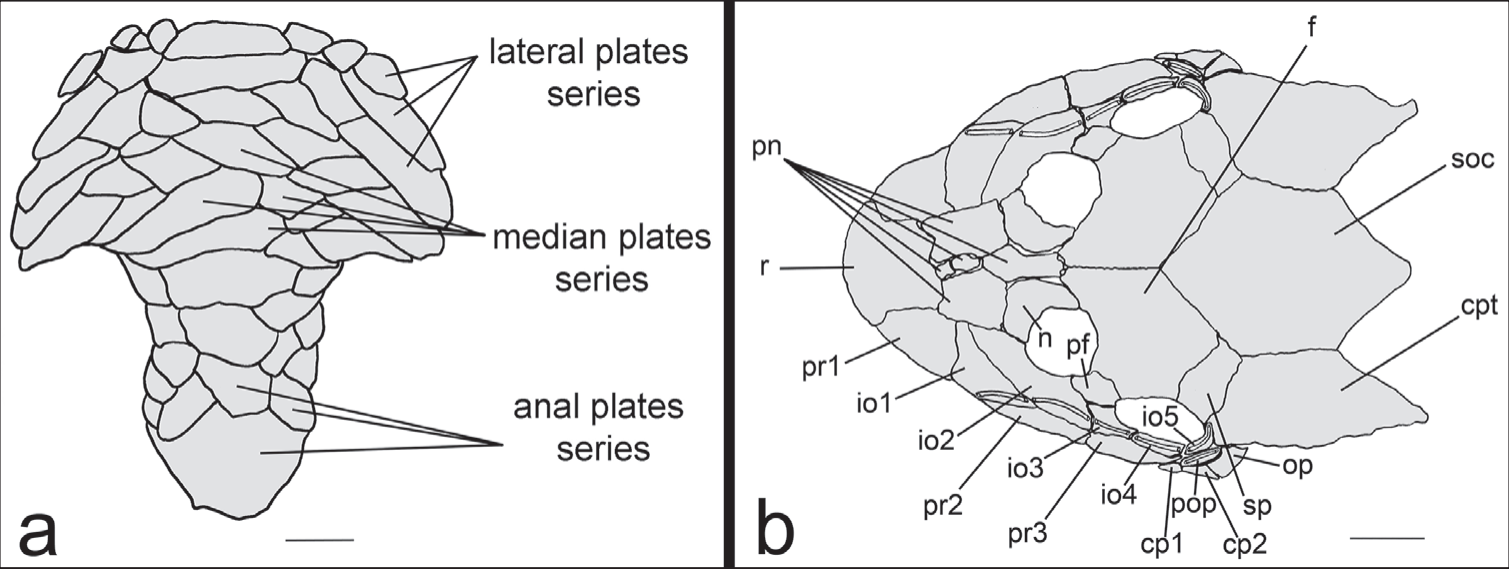
*Hisonotus acuen*, paratype, LBP 16278, 27.1 mm SL **a** Ventral view of abdominal figs **b** Cranial bones and dermal figs of the head in dorsal view. Scale bars: 1 mm.

**Figure 5.**
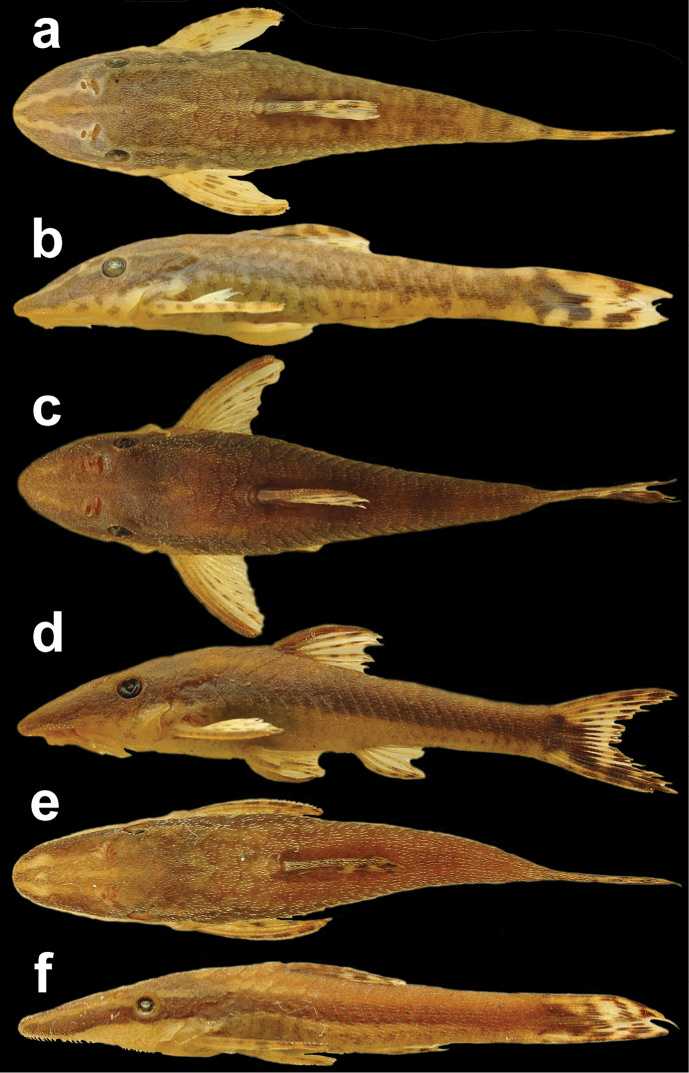
Variation in external morphology and coloration of *Hisonotus acuen*: **a, b** LBP 16279, 26.86 mm SL, affluent of rio Culuene, municipality of Gaúcha do Norte **c, d** MZUSP 115350, 25.9 mm SL, holotype, affluent of rio Toguro, municipality of Querência **e** (f) LBP16275, 21.67 mm SL, affluent of rio Feio, municipality of Querência.

*Hisonotus acuen* differs from all congeners except *Hisonotus bockmanni*, *Hisonotus chromodontus*, *Hisonotus insperatus*, *Hisonotus luteofrenatus*, *Hisonotus oliveirai* and *Hisonotus paresi* by having a functional V-shaped spinelet, Fig. 2a–f (*vs.* non-functional spinelet, a square ossification, or spinelet absent, Fig. 2g–h). It differs from *Hisonotus insperatus*, *Hisonotus paresi*, *Hisonotus luteofrenatus*, and *Hisonotus oliveirai* by having odontodes not forming longitudinally aligned rows on head and trunk (*vs.* odontodes forming longitudinally aligned rows on head and trunk). *Hisonotus acuen* differs from *Hisonotus insperatus* and *Hisonotus luteofrenatus* by the lower counts of the lateral series of abdominal figs (4−5 *vs.* 6−8 and 7−8, respectively) and from *Hisonotus insperatus* by the lower counts of the lateral median figs (22−24 *vs.* 25−26). The new species can be distinguished from *Hisonotus luteofrenatus*, *Hisonotus oliveirai* and *Hisonotus paresi* by the presence of a single rostral fig at tip of snout (*vs.* presence of a pair of rostral figs at tip of snout); from *Hisonotus bockmanni* by the absence of unpaired figlets at typical adipose fin position (*vs.* presence of the unpaired figlets); from *Hisonotus chromodontus* by having yellowish-tipped teeth, Fig. 3a (*vs.* reddish-brown teeth, Fig. 3b), by having the caudal-fin color pattern mostly hyaline, except for dark blotch on origin of rays, and dark brown chromatophores largely concentrated on rays near lower caudal spine, Fig. 3c (*vs.* caudal-fin mostly dark brown with chromatophores largely concentrated on rays and membranes, and with two hyaline spots on middle of the fin, Fig. 3d); from *Hisonotus paresi* by the absence of conspicuous dark dorsal saddle and longitudinal stripe on the body (*vs.* inconspicuous dark saddles and stripe of the body) and from *Hisonotus insperatus* by the higher number of premaxillary (14−27 *vs.* 6−12) and dentary teeth (12−23 *vs.* 5−11).

#### Description.

Morphometric and meristic data presented in Table 1. Maximum body length 29.0 mm SL. Dorsal profile of head in lateral view convex to straight from upper part of rostrum to posterior margin of nares, slightly curved from eyes to posterior margin of parieto supraoccipital, almost straight to dorsal-fin origin. Dorsal profile of trunk almost straight, descending from base of dorsal-fin origin to caudal peduncle. Ventral profile slightly concave from snout tip to anal-fin origin, slightly convex to caudal peduncle. Greatest body depth at dorsal-fin origin (13.5−22.8% SL). Greatest body width at cleithral region, gradually decreasing towards snout and caudal fin. Cross-section of caudal peduncle almost ellipsoid; rounded laterally and almost flat dorsally and ventrally.

Head rounded in dorsal view. Snout slightly pointed, its tip rounded, elongated (34.2−57.2% HL) and depressed in front of each nostril on dorsal surface. Dorsal and ventral series of odontodes completely covering anterior margin of snout; odontodes of snout similar in size to remaining ones found on head. Snout tip lacking band devoid of odontodes. Odontodes on head and trunk well defined and not forming longitudinal rows. Usually no tufts or crests of odontodes on head, in some juvenile specimens, a tiny tuft of odontodes at posterior tip of supraoccipital. Eyes small (11.2−16.2% HL), dorsolaterally positioned. Lips roundish and papillose; papillae uniformly distributed on base of dentary and premaxilla and slightly decreasing in size distally. Lower lip larger than upper lip; its border fringed. Maxillary barbel present. Teeth slender and bicuspid; mesial cusp larger than lateral cusp. Premaxillary teeth 14−27. Dentary teeth 12−23.

Dorsal-fin ii,7; dorsal-fin spinelet short and V-shaped; dorsal-fin lock functional; its origin slightly posterior to pelvic-fin origin. Tip of adpressed dorsal-fin rays slightly surpassing end of anal-fin base. Pectoral-fin i,6; tip of longest pectoral-fin ray almost reaching half of pelvic-fin length, when depressed. Pectoral axillary slit present between pectoral-fin insertion and lateral process of cleithrum. Pectoral spine supporting odontodes anteroventrally. Pelvic-fin i,5; its tip not exceeding anal-fin origin when depressed in both sex. Pelvic-fin unbranched ray with dermal flap along its dorsal surface in males. Anal fin i,5; its tip reaching 7th and 8th fig from its origin. Caudal-fin i,14,i; distal margin forked. Adipose-fin absent. Total vertebrae 27.

Body covered with bony figs except on ventral part of head, around pectoral and pelvic-fin origin and on dorsal-fin base. Cleithrum and coracoid totally exposed. Arrector fossae partially enclosed by ventral lamina of coracoids. Abdomen entirely covered by figs in adults (about 23.0 mm SL); lateral fig series with elongate and large figs, formed by two lateral fig series, similar in size; median figs formed by four to five irregular fig series reaching anal shield (Fig. 4a). Lateral side of body entirely covered by figs; mid-dorsal figs poorly developed, reaching middle of dorsal-fin base; median figs not interrupted in median portion of body, but with 2 or 3 figs not perforated before end of series; mid-ventral figs exceed end of anal-fin base.

Parts of head osteology presented in Fig. 4b. Tip of snout formed by a single almost square rostral fig (r). Nasal (n) almost rectangular, forming anterior medial nostril margin in contact posteriorly with frontals (f), and anterior and lateral margins contacting pre-nasals (pn). Lateral surface of head formed by three posterior rostrum figs (pr1-pr3) similar in size. Complete infraorbital fig series, present just below posterior rostrum series, composed of five figs (io1-io5), fourth infraorbital expanded ventrally, all associated with latero-sensory canal system; first infraorbital (io1) largest and fifth smallest (io5). Preopercle (pop) present just under fifth infraorbital (io5); an elongated bone, covered by latero-sensory canal. Subocular cheek figs (cp1-cp2) present above preopercle fig (pop). Top of head composed of compound pterotic-supracleithrum (cpt), supraoccipital (soc), prefrontal (pf), frontal (f), and sphenotic (sp); cpt covered with fenestrae randomly distributed and with different sizes and shapes.

#### Color in alcohol.

Large inconspicuous brown lateral stripe extending from tip of snout through inferior orbit to end of caudal peduncle Fig. 5a, e (very weak in some specimens, such as holotype Fig. 5c). Body ground color brown on dorsum, yellowish on ventral region under lateral stripe. Some specimens with dark saddle on mid-ventral to ventral portion of body (Fig. 5b). Dorsal, pectoral, pelvic and anal fins with brown dots on rays, varying in concentration of chromatophores from one individual to another; inter-radial membranes hyaline. Caudal fin hyaline, except for dark blotch on origin of rays, and dark brown chromatophores largely concentrated on rays near lower caudal spine (Fig. 5d). In some specimens, chromatophores forming two dark bands on middle of rays (Fig. 5b, e).

#### Color in life.

**Figure 6.**
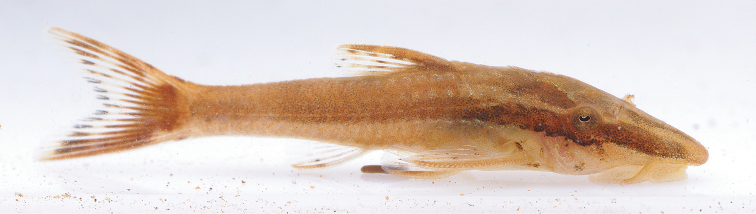
*Hisonotus acuen*, LBP 16284, live specimen, from affluent of rio Toguro, Querência, Mato Grosso State, Brazil. Photo: M. Taylor.

Similar to pattern described for alcohol individuals, but with ground color light brown (Fig. 6).

#### Sexual dimorphism.

Males bear a papilla posterior to urogenital opening and present the pelvic-fin unbranched ray with dermal flap along its dorsal surface. Both characters are absent in females.

#### Distribution.

**Figure 7.**
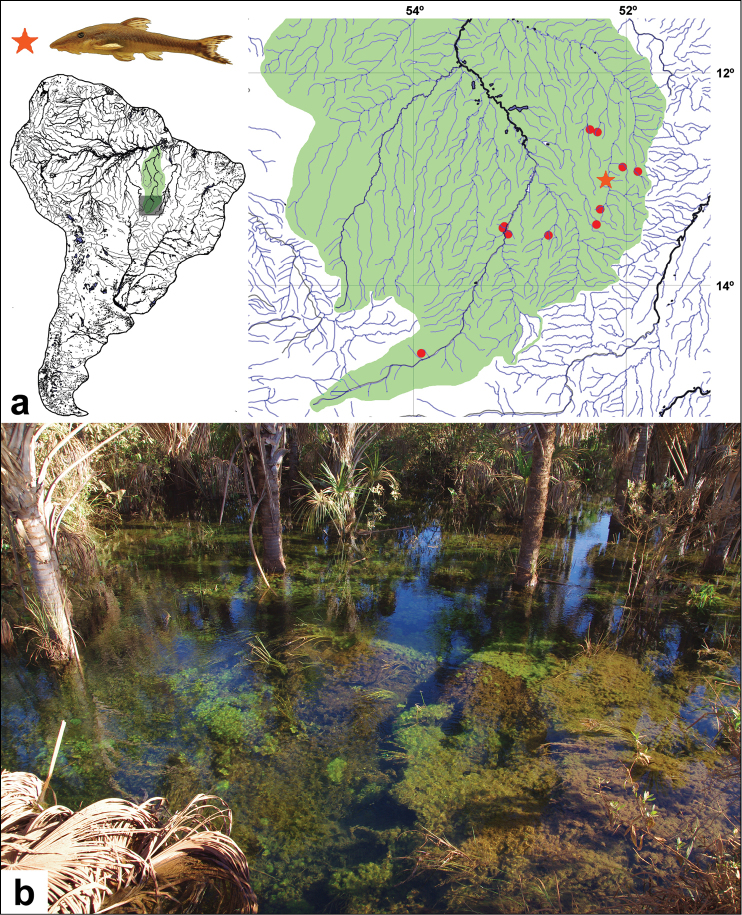
**a** Map of the distribution of *Hisonotus acuen*. Red star = holotype locality, affluent of rio Toguro. Red circles = paratype localities, affluent of rio Culuene, affluent of rio Suiá-Missu, affluent of rio Feio, córrego Xavante, rio Coronel Vanick. All are tributaries of rio Xingu, Mato Grosso State, Brazil **b** Affluent of rio Feio, municipality of Querência, 12°33'20"S, 52°16'16"W, habitat where the specimens of *Hisonotus acuen* were found. Photo: M. Taylor.

*Hisonotus acuen* is known from small to median-sized streams of the upper rio Xingu basin, Mato Grosso State in Brazil (Fig. 7a).

#### Habitat.

*Hisonotus acuen* was collected on flat areas in creeks of headwaters of the rio Xingu basin in places of shallow clear waters with low current. The fishes are found associated with vegetation that covers the bottom and the border of the headwaters (Fig. 7b).

#### Etymology.

The specific name “acuen” is in reference to the Xavante indigenous peoples, who in anthropological literature are known as “acuen”. These people are constituted by the natives inhabiting the east of the Mato Grosso State, living in the margins of the rivers Culuene, Xingu, Mortes and Araguaia.

## Discussion

The new species has a functional V-shaped spinelet (Fig. 2a–f). Carvalho and Datovo (2012) first reported this structure in *Hisonotus bockmanni*, *Hisonotus chromodontus*, *Hisonotus insperatus* and *Hisonotus luteofrenatus*. Subsequently, Roxo et al. (2014) reported this character in *Hisonotus oliveirai* and *Hisonotus paresi*. The functional V-shaped spinelet (Fig. 2a–f) is a putative apomorphic character within *Hisonotus*, and may distinguish a monophyletic group within the genus (Carvalho and Datovo 2012). However, Martins et al. (2014) have a different interpretation, in which the spinelet in *Hisonotus chromodontus*, *Hisonotus luteofrenatus* and *Hisonotus piracanjuba* is reduced, and the locking mechanism is not functional (Martins et al. 2014, Fig. 11A), the same character state found in *Hisonotus armatus*, *Hisonotus depressicauda*, *Hisonotus francirochai* and *Hisonotus notatus*. Martins et al. (2014) also suggested that in *Hisonotus insperatus* the spinelet is absent (Martins et al. 2014, Fig. 11b). However, in our interpretation, *Hisonotus acuen*, *Hisonotus chromodontus*, *Hisonotus bockmanni*, *Hisonotus insperatus*, *Hisonotus luteofrenatus*, *Hisonotus oliveirai*, *Hisonotus paresi*, and *Hisonotus piracanjuba* exhibit a functional V-shaped spinelet, which is not present in *Hisonotus depressicauda* and *Hisonotus notatus* (Fig. 2). Therefore, despite the fact that the genus *Hisonotus* may not represent a monophyletic unit, we include *Hisonotus acuen* within *Hisonotus* pending a formal phylogenetic analysis of Hypoptopomatinae, and the species-level composition is established.

**Figure 8.**
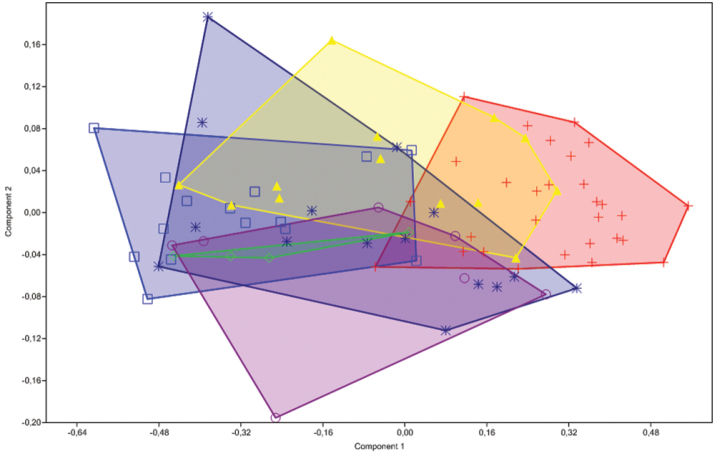
Scatter plot of Principal Component Analysis (PCA) of six allopatric populations of *Hisonotus acuen* (n = 83) indicating the presence of continuous external morphology variation. Purple circle = affluent of rio Toguro; Red cross = affluent of rio Culuene; Dark blue star = affluent of rio Suiá-Missu; Blue square = affluent of rio Feio; Yellow triangle = córrego Xavante; Green diamonds = rio Coronel Vanick. All are tributaries of rio Xingu, Mato Grosso State in Brazil.

**Figure 9.**
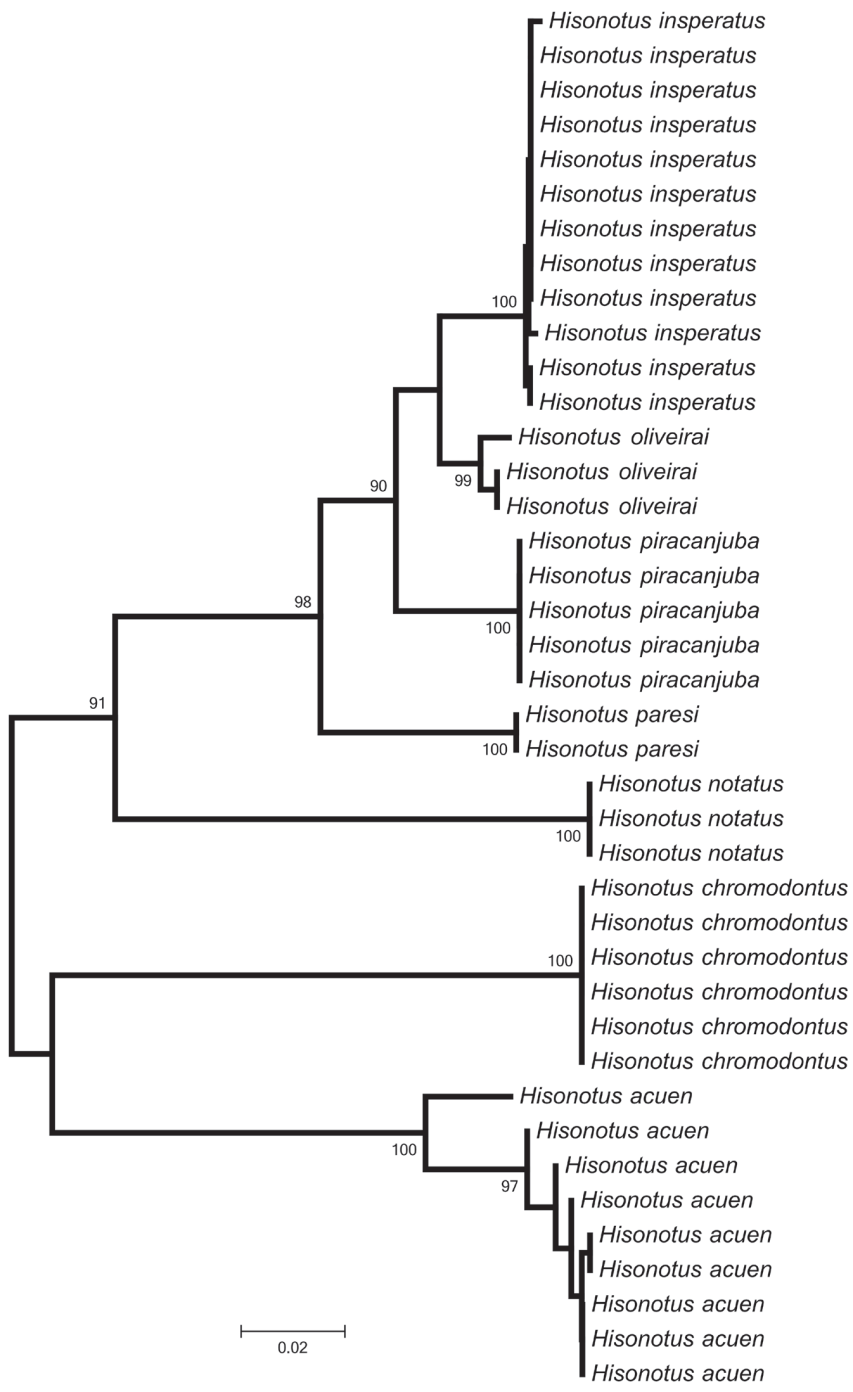
Phenogram constructed using Neighbor-Joining (NJ) method, based on the COI gene (581 pb). Numbers above branches are bootstrap values obtained from 1,000 pseudoreplicates. Values below 90% are not shown.

*Hisonotus acuen* exhibits a large amount of variation in external body proportions across its range (Fig. 5), especially in body depth at dorsal-fin origin (13.5–22.8% of SL), snout length (34.2–57.2% of HL), and abdomen length 10.2–24.4% of SL). This variation is partly distributed within populations, and partly between populations, and is not strongly correlated with body size. We performed a PCA to evaluate the morphometric variation within this new species. We compared the morphometric data of six populations found in different tributaries of the rio Xingu, and our results suggest that the range in morphology has a continuous distribution. The lack of phenotypic discontinuities among populations suggests they are not different species (Fig. 8). Additionally, we found that the genetic variation of the Cytochrome Oxidase I (COI) gene within the populations of *Hisonotus acuen* is 1%, and that variation among closely related congeners (i.e. *Hisonotus chromodontus*, *Hisonotus insperatus*, *Hisonotus oliveirai*, *Hisonotus paresi* and *Hisonotus piracanjuba*) is more than 17% (see Table 3 and Fig. 9; sequences can be downloaded from GenBank using the accession numbers provided in Methods). Therefore, the available morphological and molecular data support the recognition of the individuals inhabiting the six localities of rio Xingu and representing different populations as a single species.

**Table 3.** Genetic distance (and standard deviation) between *Hisonotus* species and specimens of the same species (main diagonal). This analysis was performed using Kimura 2-parameter substitution model, Gamma distribution and 1,000 bootstrap pseudoreplicates.

	1	2	3	4	5	6	7
1. *Hisonotus acuen*	1.0±0.2						
2. *Hisonotus paresi*	17.0%±2.3	0±0					
3. *Hisonotus piracanjuba*	21.2%±2.6	6.8%±1.3	0±0				
4. *Hisonotus chromodontus*	19.3%±2.5	19.5%±2.6	20.0%±2.6	0±0			
5. *Hisonotus notatus*	20.5%±2.8	19.0%±2.7	18.5%±2.4	22.3%±2.9	0±0		
6. *Hisonotus insperatus*	21.4%±2.7	8.0%±1.4	5.1%±0.9	21.6%±2.7	16.1%±2.1	0.1±0.1	
7. *Hisonotus oliveirai*	20.2%±2.5	6.8%±1.2	4.1%±0.9	21.2%±2.7	18.1%±2.3	3.0%±0.7	0.6±0.3

The new species *Hisonotus acuen* is the first described species of *Hisonotus* from the rio Xingu basin, and is externally very similar to *Hisonotus chromodontus*, a species from the rio Tapajos basin. The coloration of the caudal fin and the tip of the teeth distinguish these species that also are very different genetically (i.e. 19.3% of genetic divergence; Table 3 and Fig. 9). Britski and Garavello (2003) discussed the coloration of the teeth of *Hisonotus chromodontus*, reporting that in more than one hundred specimens examined, varying from 12.0 to 32.2 mm SL, all tooth-tips have a reddish-brown color. We analyzed more than one hundred specimens of *Hisonotus chromodontus* from the museum collections of LBP and NUP, and found the same reddish-brown tooth tips. This tooth features appears to be unique within the genus *Hisonotus*. A very similar external morphology, as well as the presence of the functional V-shaped spinelet among *Hisonotus acuen* and *Hisonotus chromodontus*, could suggest a close relationship between these species.

## Comparative material

All from Brazil, except when stated otherwise: *Hisonotus aky* (Azpelicueta, Casciotta, Almirón & Koerber, 2004): MHNG 2643.039, 2, 33.1−34.2 mm SL, paratypes, arroio Fortaleza, Argentina; *Hisonotus bocaiuva* Roxo, Silva, Oliveira & Zawadzki, 2013: MZUSP 112204, male, 24.2 mm SL, holotype, córrego Cachoeira, Bocaiúva, Minas Gerais; LBP 9817, 9, 3 c&s, 18.3−23.2 mm SL, paratypes, córrego Cachoeira, Bocaiúva, Minas Gerais; *Hisonotus carreiro* Carvalho & Reis, 2011: MCP 40943, 3, 33.6−35.8 mm SL, arroio Guabiju, Guabiju, Rio Grande do Sul; *Hisonotus charrua* Almirón, Azpelicueta, Casciotta & Litz, 2006: LBP 4861, 1, 35.9 mm SL, arroio Guaviyú, Artigas, Uruguai; MHNG 2650.051, 1, 34.2 mm SL, paratype, arroio Aspinillar, Uruguay; *Hisonotus chromodontus* Britski & Garavello, 2007: LBP 7964, 25, 24.0−28.3 mm SL, 3 females c&s, 26.5−28.9 mm SL, 1 male c&s 24.9 mm SL, rio dos Patos, Nova Mutum, Mato Grosso; LBP 7974, 26, 17.7–24.8 mm SL, rio dos Patos, Nova Mutum, Mato Grosso; LBP 12278, 2, 26.7−28.7 mm SL, 1 unsexed c&s, 26.7 mm SL, rio Sumidouro, Tangará da Serra, Mato Grosso; MZUSP 45355, 25.9 mm SL, holotype, affluent of rio Preto, Diamantino, Mato Grosso; MZUSP 70758, 7, 19.4−23.9 mm SL, paratype, riacho Loanda, Sinop, Mato Grosso; NUP 10924, 24, 19.5−31.5 mm SL, rio Preto, Diamantino, Mato Grosso; *Hisonotus depressicauda* (Miranda Ribeiro, 1918): MZUSP 5383, 24.4 mm SL, paralectotype, Sorocaba; LBP 17474, 5 c&s, 18.1−24.0 mm SL, rio Araquá, Botucatu, São Paulo; *Hisonotus francirochai* (Ihering, 1928): LBP 13923, 22, 25.7−35.7 SL, córrego sem nome, Capitinga, Minas Gerais; MZUSP 3258, 29.4 mm SL, lectotype, rio Grande, São Paulo; *Hisonotus heterogaster* Carvalho & Reis, 2011: LBP 3335, 39, 20.8−30.1 mm SL, arroio sem nome, rio Grande, Rio Grande do Sul; *Hisonotus insperatus* Britski & Garavello, 2003: LBP 1299, 3, 23.5−29.6 mm SL, 1 female c&s, 24.8 mm SL, rio Araquá, Botucatu, São Paulo; LBP 1316, 2, 24.1−27.4 mm SL, 1 female c&s, 24.7 mm SL, 1 male c&s, 23.9 mm SL, rio Araquá, Botucatu, São Paulo; LBP 1344, 2, 22.9−24.9 mm SL, rio Araquá, Botucatu, São Paulo; LBP 1373, 1, 25.8 mm SL, rio Araquá, Botucatu, São Paulo; LBP 1405, 2, 22.2−27.3 mm SL, rio Araquá, Botucatu, São Paulo; LBP 4699, 17, 19.6−26.9 mm SL, 4 females c&s, 20.3−26.8 mm SL, 3 males c&s, 24.3−26.1 mm SL, ribeirão Cubatão, Marapoama, São Paulo; LBP 4945, 5, 27.3−28.5 mm SL, 2 females c&s, 28.2−29.9 mm SL, Botucatu, São Paulo; LBP 6770, 5, 25.1−28.2 mm SL, 3 females c&s, 20.0−27.0 mm SL, ribeirão Cubatão, Marapoama, São Paulo; LBP 13336, 1 female c&s, 26.0 mm SL, rio Capivara, Botucatu, São Paulo; LBP 13337, 2 females c&s, 27.4−28.6 mm SL, rio Araquá, Botucatu, São Paulo; MZUSP 22826, 1, 25.4 mm SL, paratype, córrego Água Tirada, Três Lagoas, Mato Grosso; MZUSP 24832, 1, 23.8 mm SL, paratype, rio Corumbataí, Corumbataí, São Paulo; MZUSP 78957, 29.6 mm SL, holotype, rio Capivara, Botucatu, São Paulo; MZUSP 78960, 31, 12.6−26.0 mm SL, paratypes, 5 c&s, 22.7−24.7 mm SL, rio Pardo, Botucatu, São Paulo; MZUSP 78965, 10, 15.6−28.6 mm SL, paratypes, 3 c&s, not measured, rio Araquá, Botucatu, São Paulo; MZUSP 78968, 5, 24.1−27.3 mm SL, paratypes, córrego da Figueira, Lins, São Paulo; *Hisonotus iota* Carvalho & Reis, 2009: LBP 13072, 5, 32.3−33.0 mm SL, rio Chapecó, Coronel Freitas, Santa Catarina; *Hisonotus laevior* Cope, 1894: LBP 3377, 1, 25.2 mm SL, arroio dos Corrientes, Pelotas, Rio Grande do Sul; LBP 6037, 8, 33.4−47.0 mm SL, rio Maquiné, Osório, Rio Grande do Sul; LBP 13187, 7, 19.4−45.8 mm SL, córrego sem nome, Camaquá, Rio Grande do Sul; *Hisonotus leucofrenatus* (Miranda Ribeiro, 1908): LBP 2085, 7, 38.3−50.6 mm SL, rio Sagrado, Morretes, Paraná; LBP 6837, 36, 35.1−43.5 mm SL, rio Fau, Miracatu, São Paulo; *Hisonotus leucophrys* Carvalho & Reis, 2009: LBP 13065, 6, 17.2−33.6 mm SL, rio Ariranhas, Xavantina, Santa Catarina; LBP 13073, 1, 36.8 mm SL, rio Guarita, Palmitinho, Rio Grande do Sul; *Hisonotus luteofrenatus* Britski & Garavello, 2007: MZUSP 62593, 28.6 mm SL, holotype, córrego Loanda, Cláudia, Mato Grosso; MZUSP 62594, 8, 22.4−30.5 mm SL, paratype, riacho Selma, Sinop, Mato Grosso; MZUSP 95940, 3, 26.1−28.5 mm SL, affluent of rio Teles Pires, Itaúba, Mato Grosso; *Hisonotus megaloplax* Carvalho & Reis, 2009: LBP 13108, 6, 36.4−37.8 mm SL, córrego sem nome, Saldanha Marinho, Rio Grande do Sul; *Hisonotus montanus* Carvalho & Reis, 2009: LBP 13051, 3, 26.4−27.2 mm SL, rio Goiabeiras, Vargem, Santa Catarina; LBP 13055, 5, 24.8−31.9 mm SL, rio Canoas, Vargem, Santa Catarina; *Hisonotus nigricauda* (Boulenger, 1891): LBP579, 16, 34.1−40.1 mm SL, rio Guaíba, Eldorado do Sul, Rio Grande do Sul; *Hisonotus notatus* Eigenmann & Eigenmann, 1889: LBP 3472, 20, 21.0−34.3 mm SL, 2 males c&s 25.8−26.5 mm SL, 1 female c&s, 25.0 mm SL, rio Aduelas, Macaé, Rio de Janeiro; LBP 10742, 25, 24.4−43.3 mm SL, rio Macabu, Conceição de Macabu, Rio de Janeiro; *Hisonotus oliveirai* Roxo, Zawadzki & Troy, 2014: MZUSP 115061, female, 26.4 mm SL, holotype, ribeirão Cambira, affluent of rio Ivaí, Cambira, Paraná; LBP 13332, 1 male, 23.2 mm SL, 1 unsexed c&s, 23.7 mm SL, paratype, rio Mourão, rio Ivaí basin, Campo Mourão, LBP 17578, 3 females, 27.7−30.4 mm SL, 2 males, 25.4−26.1 mm SL, paratypes, rio Mourão, rio Ivaí basin, boundary between Engenheiro Beltrão and Quinta do Sol; NUP 3578, 7 females, 27.8−28.1 mm SL, 8 males, 24.7−26.8 mm SL, 1 female c&s, 27.6 mm SL, 1 male c&s, 25.5 mm SL, paratypes, ribeirão Salto Grande, rio Ivaí basin, Maria Helena; *Hisonotus paresi* Roxo, Zawadzki & Troy, 2014: MZUSP 115062, female, 26.2 mm SL, holotype, riacho Águas Claras, affluent of rio Sepotuba, Santo Afonso; LBP 13351, 9, 14.7−24.3 mm SL, paratype, riacho Águas Claras, Santo Afonso; LBP 13352, 1, 23.7 mm SL, paratype, riacho Águas Claras, Santo Afonso; NUP 10928, 2 males, 23.2−24.2 mm SL, paratype, 2 c&s, 23.6−24.2 mm SL, riacho Águas Claras, afluente of rio Sepotuba, Santo Afonso; NUP 10976, 3 unsexed, 16.7−20.5 mm SL, paratype, riacho São Jorge, Santo Afonso; *Hisonotus piracanjuba* Martins & Langeani, 2012: LBP 17256, 9, 17.2−26.3 mm SL, 1, c&s 27.1 mm SL, córrego sem nome, Morrinhos, Goiás; NUP 5059, 1, 24.7 mm SL, córrego Posse, Anápolis, Goiás; NUP 10979, 3, 21.4−21.8 mm SL, ribeirão Bocaina, Piracanjuba, Goiás; *Hisonotus prata* Carvalho & Reis, 2011: MCP 40492, 18, 19.5−33.2 mm SL, rio da Prata, Nova Prata, Rio Grande do Sul; LBP 9918, 14, 21.7−32.6 mm SL, Laguna dos Patos system, Nova Prata, Rio Grande do Sul; *Hisonotus ringueleti* Aquino, Schaefer & Miquelarena, 2001: FMNH 108806, 2, 25.7−32.2 mm SL, rio Quaraí basin, Uruguay; LBP 13148, 1, 24.5 mm SL, arroio Putiá, Uruguaiana, Rio Grande do Sul; *Hisonotus* sp.: LBP 8276, 1 c&s, 25.6 mm SL, rio Verde Grande, Jaíba, Minas Gerais; *Microlepidogaster arachas* Martins, Calegari & Langeani, 2013: LBP 10882, 3, 22.8−35.3 mm SL, rio Paraná basin, Araxás, Minas Gerais; *Microlepidogaster dimorpha* Martins & Langeani, 2011: LBP 10683, 2, 28.8−35.6 mm SL; rio Paraná basin, Uberaba, Minas Gerais; *Otothyris travassosi* Garavello, Britski & Schaefer, 1998: LBP 1971, 13, 14.0−27.2 mm SL; coastal drainage, Canavieiras, Bahia; *Otothyropsis marapoama* Ribeiro, Carvalho & Melo, 2005: LBP 4698, 6, 23.9−36.3 mm SL; rio Tietê basin, Marapoama, São Paulo; *Parotocinclus aripuanensis* Garavello, 1988: LBP 10981, 33, 15.63−18.47 mm SL, rio Lageado, Guajará Mirim, Rondônia; Parotocinclus aff. spilurus (Fowler, 1941): LBP 5624, 21.5−22.0 mm SL, rio Maravilha, Balsas, Maranhão; Parotocinclus cf. bahiensis (Miranda Ribeiro, 1918): LBP 7182, 3, 27.9−35.6 mm SL; rio Paraguaçu basin, Lençóis, Bahia; *Parotocinclus maculicauda* (Steindachner, 1877): LBP 2869, 15, 20.2−44.7 mm SL, rio Ribeira do Iguape basin, Miracatu, São Paulo; *Parotocinclus prata* Ribeiro, Melo & Pereira, 2002: LIRP 1136, 38, 19.8−41.9 mm SL; rio São Francisco basin, Presidente Olegário, Minas Gerais; *Parotocinclus* sp.: LBP 1572, 3, 19.0−24.0 mm SL, ribeirão Ínsula, Barra do Garça, Mato Grosso; LBP 2414, 19, 16.7−20.8 mm SL, 1 c&s 23.6 mm SL, córrego Fundo, Barra do Garça, Mato Grosso.

## Supplementary Material

XML Treatment for Hisonotus acuen
